# Dicarbonyl Stress at the Crossroads of Healthy and Unhealthy Aging

**DOI:** 10.3390/cells8070749

**Published:** 2019-07-19

**Authors:** Cecilia Nigro, Alessia Leone, Francesca Fiory, Immacolata Prevenzano, Antonella Nicolò, Paola Mirra, Francesco Beguinot, Claudia Miele

**Affiliations:** 1 URT Genomic of Diabetes, Institute of Experimental Endocrinology and Oncology, National Research Council, 80131 Naples, Italy; 2 Department of Translational Medicine, Federico II University of Naples, 80131 Naples, Italy

**Keywords:** aging, diabetes mellitus, dicarbonyl stress, glycation, glyoxalase system, hormesis, methylglyoxal, neurodegeneration, obesity, vascular complications

## Abstract

Dicarbonyl stress occurs when dicarbonyl metabolites (i.e., methylglyoxal, glyoxal and 3-deoxyglucosone) accumulate as a consequence of their increased production and/or decreased detoxification. This toxic condition has been associated with metabolic and age-related diseases, both of which are characterized by a pro-inflammatory and pro-oxidant state. Methylglyoxal (MGO) is the most reactive dicarbonyl and the one with the highest endogenous flux. It is the precursor of the major quantitative advanced glycated products (AGEs) in physiological systems, arginine-derived hydroimidazolones, which accumulate in aging and dysfunctional tissues. The aging process is characterized by a decline in the functional properties of cells, tissues and whole organs, starting from the perturbation of crucial cellular processes, including mitochondrial function, proteostasis and stress-scavenging systems. Increasing studies are corroborating the causal relationship between MGO-derived AGEs and age-related tissue dysfunction, unveiling a previously underestimated role of dicarbonyl stress in determining healthy or unhealthy aging. This review summarizes the latest evidence supporting a causal role of dicarbonyl stress in age-related diseases, including diabetes mellitus, cardiovascular disease and neurodegeneration.

## 1. Introduction—Dicarbonyl Stress and Glycation

Dicarbonyl stress is a dysfunctional state where methylglyoxal (MGO) and other reactive α-oxaldehyde metabolites accumulate as consequence of their increased formation or decreased activity of the detoxifying systems [1]. Typical concentrations of MGO, glyoxal (GO) and 3-deoxyglucosone (3-DG) have been estimated to be 50–150 nM in human plasma and 1–4 µM in human cells [2]. MGO is the most reactive dicarbonyl and of the highest endogenous flux (ca. 3 mmol per day) [3]. Therefore, it is commonly the primary concern. 

The major source of MGO formation is the spontaneous degradation of triosephosphates, DHAP (dihydroxyacetone phosphate) and GA3P (glyceraldehyde 3-phosphate), intermediates of the glycolysis pathway, but also of gluconeogenesis and glyceroneogenesis. A range of 0.05%–0.1% of glucose triose flux contributes to MGO formation [4], which is heightened by: increased glucose metabolism in hyperglycemia associated with diabetes, impaired disposal of GA3P by a decreased activity of the reductive pentose phosphate pathway, and increased dependence on anaerobic glycolysis and related increased metabolic flux in hypoxia [1].

Other minor sources of MGO are: 1. the degradation of proteins glycated by glucose, whose contribution is ca. 10% of total MGO exposure [5]; 2. threonine catabolism from the oxidation of aminoacetone by the amino oxidase [6], contributing to ca. 3% of total MGO exposure; 3. the ketone body metabolism, where acetone derives from the degradation of acetoacetate and is hydroxylated to hydroxyacetone, which is further oxidized to form MGO [7]. The latter pathway may give a relevant contribution to MGO levels in conditions favoring ketone bodies’ accumulation, such as the consumption of a diet rich in fats or diabetic ketoacidosis. Although foodstuffs contain variable levels of MGO, the contribution of exogenous sources is low (<1% of total MGO exposure) and still controversial, as ingested MGO appears to be metabolized pre-systemically and rapidly reacts in the intestinal lumen before adsorption [8].

Besides the negligible amount of exogenous MGO, it is endogenously formed into the cells and may diffuse through the interstitial fluids into the plasma and to other cells [1]. In physiological solutions, MGO exists as unhydrated (1%) and mono- or di-hydrated forms (70% and 29%, respectively), which are rapidly interconverted [9]. The unhydrated hydrophobic form is able to cross the cell membrane by passive diffusion, and has an estimated half-life of ca. 4 minutes [10]. Most glycation reactions of MGO occur in the unhydrated form [11], and this probably accounts for its short half-life. The percentage of MGO in its unhydrated form is hundreds of times higher than GO [12], which makes MGO the most reactive dicarbonyl. 

Glycation by MGO produces intra- and extra-cellular modification at similar or even higher levels than that by glucose [13]. Ninety-nine percent of MGO rapidly and reversibly binds to cysteine thiols forming hemithioacetal residues. The irreversible reaction of MGO with proteins is directed to arginine and lysine residues, forming advanced glycated end products (AGEs). The hydroimidazolone adduct (MG-H1), derived from the binding of MGO to arginine, is the most quantitatively (>90% of MGO adducts) and functionally important AGE in physiological systems. Dicarbonyl glycation has important functional implications, as arginine has a high probability of location in the functional sites of proteins [14] and its glycation induces loss of positive charge, which means loss of electrostatic interactions involved in the side-chain guanidine group and ligand-binding interactions, resulting in protein inactivation and dysfunction [15]. MGO also forms DNA adducts, of which imidazopurinone derivate 3’-(2’-deoxyribosyl)-6,7-dihydro-6,7-dihydroxy-6-methylimidazo-[2,3-b]purine-9(8)one (MGdG), is a quantitatively major nucleotide AGE [9]. MGO-derived nucleotide adducts are associated with DNA single-strand breaks and increased mutation frequency. A frequency of ca. 9 adducts per 106 nucleotides [16] and one MG-H1 residue per 1%–5% protein [9] has been estimated in the steady state *in vivo*, which increases in aging and disease associated with dicarbonyl accumulation. AGEs are continuously formed in mammal organisms during life and are recognized as reliable markers of age-related changes. Very recently, Bilova et al. have indicated the existence of age-related glycation hot-spots also in the plant proteome [17].

MGO and GO are metabolized mainly by the glyoxalase system, with minor metabolism by aldoketo reductases (AKRs) and aldehyde dehydrogenases (ADHs). However, glyoxalase activity was estimated to exceed that of AKR for MGO metabolism by >30-fold in human tissue [18]. Whilst, ADH-linked MGO metabolism was almost undetectable [19]. Glyoxalase system is a cytoplasmic enzyme system, consisting of glyoxalase 1 (Glo1), glyoxalase 2 (Glo2) and a catalytic amount of glutathione (GSH). It is present in all mammalian cells and catalyses the metabolism of most of MGO to D-lactate [20]. It has therefore been defined as the major dicarbonyl stress defense system.

As the result of MGO formation/detoxification balance alteration, dicarbonyl stress is fostered by a reduction in the expression or activity of Glo1. Glo1 activity is directly proportional to its cofactor GSH concentration, therefore oxidative or non-oxidative depletion of GSH favors dicarbonyl stress [21]. Basal and inducible expression of Glo1, as well as AKRs and ADH, are under stress-responsive control by nuclear factor erythroid 2-related factor (Nrf2) binding to antioxidant response elements (AREs) [22]. Dicarbonyls themselves may bind to reactive cysteine residues in the regulatory inhibitory protein of Nrf2, kelch-like ECH-associated protein 1 (Keap1), by disrupting the nuclear translocation of Nrf2, necessary to the transcriptional activation of Glo1 [23]. Furthermore, the activation of the nuclear factor kappa-light-chain-enhancer of activated B cells (NF-ĸB) system in inflammation and downstream of the AGE/receptor of AGE (RAGE) pathway [24] inhibits Nrf2, therefore downregulating Glo1 expression. This is also negatively regulated by HIF1-α (hypoxia-inducible factor 1-α) in hypoxia [25], which is an important driver of dicarbonyl stress by both increasing MGO formation through anaerobic glycolysis and inhibiting Glo1 expression.

Reduced activity of Nrf2 and increased oxidative stress in aging and disease may predispose to dicarbonyl stress, which is beginning to feature strongly as a driver of pathogenesis in aging-related disease.

## 2. Hormesis: A First Line of Cellular Defense to Counteract Dicarbonyl Stress

One dark side of energy production is the accumulation of toxic metabolites, which contribute to the aging process. During evolution, cells have developed an adaptive response mechanism to restore the disrupted homeostasis and prevent the damage induced by toxic agents [26]. This mechanism is known as hormesis and defines a general phenomenon whereby a mild stress-induced stimulation results in biologically beneficial effects. These are associated with higher levels of stress tolerance than the prior stage, whereas cell death represents a final process where failure in adaptation or unhealthy adaptation occurs [27].

Many evidence have been collected on the hormetic effect exerted by reactive oxygen species (ROS). Oxidative stress is one of the major cause of aging and many observations show that lowering the burden of oxidative stress leads to lifespan extension [28,29]. However, a mild increase in ROS levels may rather have beneficial effects on health and lifespan by inducing mitochondrial hormesis, also called mitohormesis, and improving systemic defense mechanisms like control of proteostasis, unfolded protein response (UPR) and stress resistance [30].

Very recent studies have provided major evidence that a hormetic effect is exerted also by reactive carbonyl species (RCS), indicating that next to mitohormesis there also exists “glycohormesis”. Zemva et al. have demonstrated in yeast that low levels of MGO activate a multi-layered defense response [31]. This feedback mechanism includes a transcriptional response aimed at the prevention of RCS enhanced production, detoxification of reactive metabolites (i.e., induction of Nrf2 and upregulation of Glo1) and remission of damage by the protein quality control system (i.e., induction of molecular chaperones HSP70 and BTN2). The latter is involved in handling or sorting MGO-modified proteins to specialized cellular protein deposition sites, therefore directly linking metabolic to proteotoxic stress. The glycohormetic response also exists in mammalian cells and enables them to pre-adapt to rising energy flux [31].

Although a previous functional genomic study published by Morcos et al. [32] demonstrated that the age-related decrease in Glo1 and the concomitant increase in mitochondrial MGO-derived AGEs are crucial in reducing lifespan in *C. Elegans*, it has been more recently demonstrated a hormetic modulation of lifespan by MGO [33]. Indeed, the dose-response experiments performed by Ravichandran et al. in *C. Elegans* did not result in a linear but in a J-shaped curve, where at low MGO concentration (≤100 µM) they observed a lifespan increase and, at higher MGO concentration (≥1 mM) a reduction in lifespan. Dicarbonyls have been previously proposed to reduce proteasome activity, thus impairing health and potentially lifespan [34]. This study has shown that low doses of MGO induce proteasome activity, indicating a non-linear response to be called proteohormesis [33]. The beneficial effect of low-dose MGO on the nematode healthspan is mediated by the activation of the stress response and ubiquitin-proteasome system, specifically by Skinhead-1 (SKN-1)/Nrf2 and heat shock factor 1 (HSF-1) regulation.

These two transcription factors are also implicated in the hormetic stress of the worms in response to phytochemicals, that at low-dose activate an adaptive stress-response resulting in the lifespan extension [35]. As the result of SKN-1/Nrf2 activation, hormetic stress also resists to glucose toxicity by enhancing the Glo1 expression and reducing MGO accumulation in *C. Elegans* [35].

An analogous lifespan extension described by Ravichandran et al. [33] was found by Moraru et al. in a transgenic model of *Drosophila* with a similar elevation of MGO levels, suggesting that this is an evolutionarily conserved response [36]. The explanation of this effect found by Moraru et al. was the reduced insulin signaling implemented by the organism as a feedback mechanism to reduce glucose uptake and triose phosphate production.

A hormetic effect exerted by AGEs was also reported by Fabre et al. [37] on the liver of rats. Although considered toxic, this study shows that AGEs become protective when chronically administrated by stimulating the protein kinase B (AKT) signaling, which is involved in both cellular defense, by promoting the Nrf2 nuclear translocation, and insulin sensitivity.

Therefore, in response to dicarbonyls and the deriving AGEs, cells adopt a bi-phasic hormetic phenomenon where a first mild exposure leads to the activation of a defense mechanism that increases the cell resistance to a later greater stress; whereas, at higher levels of RCS, such as in abnormal metabolic conditions, cell function and viability are under threat.

## 3. Dicarbonyl Stress in Aging

The aging process implicates a progressive and irreversible damage to intra- and extra-cellular macromolecules, resulting by the reduced or impaired glycation and oxidative defense mechanisms.

Dicarbonyl stress contributes to aging through the age-related decline in Glo1. Glo1 activity was found to be decreased in arterial tissue, human lens and brain with aging [38]. MGO-derived AGEs were reported to be increased in aging tissues, contributing to macular degeneration in the retina [39], osteoarthritis in human cartilage [40], impaired endothelium-dependent vasorelaxation in vascular tissue [41], endoplasmic reticulum (ER) stress and apoptosis in skin fibroblasts [42] and skin aging [43].

The first causal link between AGEs and aging was provided by Morcos et al. [32] in *C. Elegans*, demonstrating that an age-related decrease in Glo1 activity increases mitochondrial ROS production, thereby limiting lifespan. Consistently, the overexpression of Glo1 prevents the accumulation of MG-H1 residues in mitochondrial proteins, decreasing ROS production and increasing the lifespan of the nematode.

**Figure 1:**
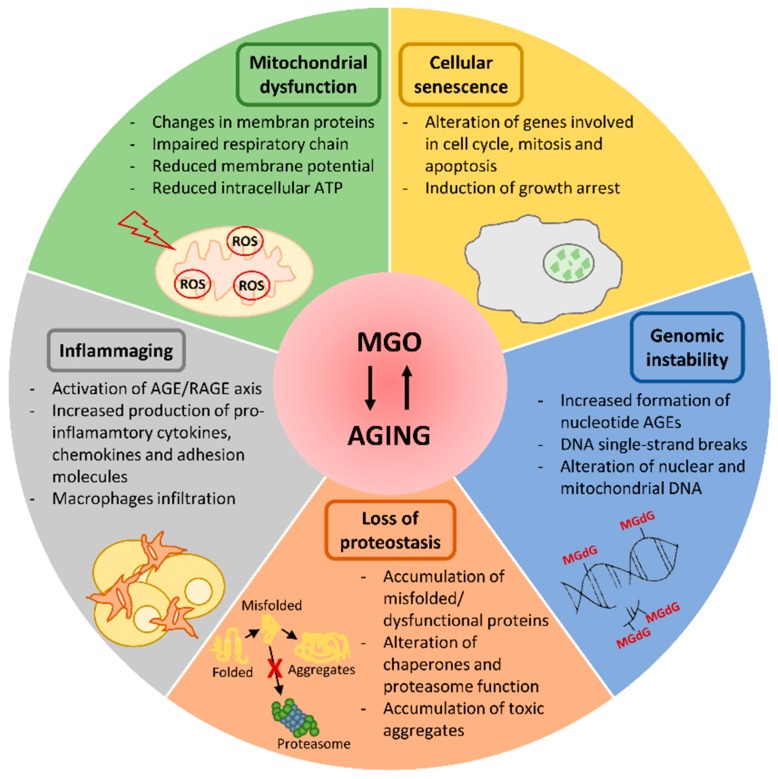
Cellular processes compromised by methylglyoxal (MGO) and aging. MGO accumulation fosters some aging-related molecular events that are schematically reported in the figure. AGE: advanced glycation end products; MGdG: 3’-(2’-deoxyribosyl)-6,7-dihydro-6,7-dihydroxy-6-methylimidazo-[2,3-b]purine-9(8)one; RAGE: advanced glycation end products receptor.

The aging process is characterized by the progressive loss of physiological functions, which leads to enhanced risk for major pathologies, namely diabetes mellitus (DM), cardiovascular disease (CVD), neurodegeneration and cancer. On a molecular level various hallmarks have been reported by Lopez-Otin et al. in 2013 [44] to explain the aging process. Many of these aging-related molecular events can be fostered by dicarbonyl accumulation, which thus represents a determinant of the aging phenotype. Dicarbonyl stress contribution to the alteration of some aging-determining biochemical processes is described in the following (Figure 1).

### 3.1. Mitochondrial Dysfunction

Mitochondrial proteins are considered the major targets of dicarbonyl glycation. The higher glycation of these proteins is associated with an increased production of ROS and proteome damage [45]. ROS have a harmful effect on cellular homeostasis because of their capacity to interact with proteins, lipids and DNA, thus altering their structure and functions [46]. In the mitochondrion, excess of ROS leads to changes in mitochondrial permeability, an index of mitochondrial dysfunction [47], and contributes to the development of several age-related diseases [48].

MGO directly modifies mitochondrial membrane proteins and antioxidant enzymes [49], while Glo1 overexpression is able to decrease dicarbonyl glycation of mitochondrial proteins and ROS production. This was found to be associated with life extension in *C. elegans* [45].

A study performed in cultured vascular smooth muscle A-10 cells has shown that mitochondrial function is damaged at several levels by MGO treatment. MGO-induced glycation of mitochondrial proteins leads to an accumulation of carboxyetil-lysine (CEL), and increases the generation of ROS, peroxynitrite, mitochondrial superoxide and nitric oxide (NO). Furthermore, MGO reduces the activity of manganese superoxide dismutase (mnSOD), which catalyzes superoxide degradation, representing the first line of defense against oxidative stress [50], and reduces complex III activity and production of adenosine triphosphate (ATP), thus impairing the mitochondrial respiratory chain [50]. This last effect exerted by MGO on mitochondria has also been demonstrated in SH-SY5Y neuroblastoma cells and in retinal pigment epithelial cells, where MGO induces cellular cytotoxicity, characteristic of neurodegeneration and age-related macula degeneration in neurons and retinal cells, respectively. In these cell lines, it has been demonstrated that MGO compromises mitochondrial integrity by decreasing mitochondrial membrane potential and intracellular ATP levels [51,52].

In the last few years, several strategies have been proposed to ameliorate the damage induced by MGO on mitochondria. Among these, Seo et al. demonstrated that resveratrol (RES), a polyphenolic component found in grapes and red wine, already known to have many beneficial effects on metabolic diseases and aging, is able to prevent the human hepatocyte carcinoma (HepG2) cell death caused by MGO-induced mitochondrial dysfunction [47]. In detail, RES treatment induces the expression of Sestrin 2 (SESN2), a novel antioxidant protein that inhibits mitochondrial dysfunction and apoptosis of these cells [47].

Moreover, high MGO levels affect osteoblastic function in MC3T3-L1 cells by reducing osteoblast differentiation and inducing osteoblast cytotoxicity. These effects are induced by an increase of intracellular ROS, mitochondrial superoxide and cardiopilin peroxidation formation [53]. Suh et al. have recently indicated piceatannol, an analogue of RES, as natural molecule able to ameliorate MGO-induced mitochondrial dysfunction by inhibiting inflammatory cytokines and ROS production [54].

Beyond mitochondrial proteins, also mitochondrial DNA (mtDNA) is a target for glycation. As demonstrated by Breyer et al., mtDNA is more vulnerable to glycation than nuclear DNA. Indeed, in cultured fibroblasts, the amount of the major nucleotide glycation adduct N2 -carboxyethyl-20 -deoxyguanosine (CEdG) was found to be increased up to three fold in mtDNA compared to nuclear DNA [55]. The effect of dicarbonyl stress-induced nucleotide adducts are further described below.

### 3.2. Loss of Proteostasis

Proteostasis includes cellular processes involved in the stabilization of proteins, such as chaperone-assisted protein folding, and those involved in protein degradation, such as the proteasome or lysosome [56,57]. Impaired proteostasis is a feature of aging and some aging-related disease [44], including neurodegeneration, metabolic and vascular dysfunction. The effect of MGO on proteins is the formation of AGEs that are resistant to proteolysis [58]. Recovery from dicarbonyl stress and replacement of proteins damaged by dicarbonyl glycation involves the proteasomal system. Therefore, the age-related loss of proteasomal activity is linked to the accumulation of damaged proteins, which may act itself as a cause of reduced proteasomal capacity and increased risk of irreversible tissue damage and cell senescence. Among the chaperone proteins, the heat shock proteins (HSP) are known to be targets of MGO modification. HSP27 is the major HSP protein modified by MGO in several cell types [59,60] and tissues [61,62].

The accumulation of altered proteins can also be related to a decreased ability of intracellular proteases to degrade aberrant polypeptide chains [63]. MGO modifies the 20S proteasome, decreasing its activity and reducing the polyubiquitin receptor 19S-S5a in mouse diabetic kidney [34]. Moreover, it has been demonstrated in human retinal pigment epithelial (ARPE-19) cells that MGO accumulation impairs both the ubiquitin-proteasome system (UPS) and the protein quality control dependent on C-terminus of HSC70-interacting protein (CHIP) and molecular chaperones, inducing the accumulation of toxic aggregates and increasing cell death [64].

Therefore, counteracting dicarbonyl stress becomes an essential target to guarantee the cellular proteostasis and, thus, a healthy aging.

### 3.3. Cellular Senescence

Increased levels of GO and MGO observed in diabetes and aging are biologically relevant as they induce cell senescence in human vascular endothelial cells (ECs). A study by Santos et al. demonstrated that a combination of GO and MGO is able to increase p21 expression and to arrest human vascular ECs in the G2-phase of the cell cycle, besides the increase of ROS and AGEs formation [65], which can be abrogated by antioxidant and dicarbonyl scavenger treatment [65].

Similarly, in human umbilical vein ECs (HUVECs) treated with tumor necrosis factor (TNF)-alpha, high MGO levels severely alter the gene expression profile by affecting genes involved in cell cycle, mitosis and apoptosis. Among these, several genes belonging to p53 signaling pathway results significantly modified by MGO. In detail, those genes inversely associated with p53 activation (i.e., cyclin-dependent kinase 1, cyclin B2, cyclin B1, cyclin G2) are down-regulated by MGO; conversely, those genes involved in apoptosis, growth arrest or p53 activation (i.e., insulin-like growth factor binding protein 3, sestrin 2, p21) are upregulated. This indicates that MGO treatment induces HUVECs’ cell growth arrest and apoptosis-mediated cytotoxicity [66]. Carnosine treatment has been proposed as a strategy to ameliorate the harmful effect of MGO on HUVECs. It has already been demonstrated that carnosine can directly react with MGO, exerting a protective effect against macromolecule damage in the brain, liver and kidney [63]. Furthermore, carnosine counteracts gene expression changes induced by MGO in HUVECs [66].

### 3.4. Inflammaging

During mammal aging, a pro-inflammatory phenotype can derive from the activation of several conditions such as: the dysfunctional immune system, the increased production of pro-inflammatory cytokines by senescent cells, the increased activation of some transcriptional factors (i.e., NF-ĸB) and the reduction of autophagy response [67]. This phenotype represents an important aging-associated alteration in intracellular communication and is known as “inflammaging” [44].

AGEs and their dicarbonyl precursors, such as MGO, play a pivotal role in the development and progression of aging-related chronic disease. During normal aging, they mainly accumulate slowly in the human body but are also introduced with diet [68]. It is well established that accumulation of endogenous AGEs induce the activation of pro-inflammatory mechanisms typical of several pathological conditions, but about exogenous AGEs activity and metabolic fate, little is known yet [68]. In a streptozotocin (STZ)-induced diabetic mouse model, Lv et al. demonstrated that a high-AGEs diet worsen the pro-inflammatory profile and renal and heart complications of these mice [69].

The activation of AGE–RAGE axis is currently considered the principal mechanism by which AGEs activate inflammatory processes. Following AGEs binding to RAGE, nicotinamide adenine dinucleotide phosphate (NADPH) oxidase increases ROS production that in turn activates NF-ĸB. Once activated, NF-ĸB induces the transcription of several pro-inflammatory cytokines (TNF-α, IL-6), chemokines (MCP-1), adhesion molecules (E-selectine, ICAM-1) and others [68,70]. 

Recent studies have demonstrated the beneficial role of natural compounds on the progression of age-related pathologies, thanks to their anti-inflammatory and anti-oxidative properties. In cultured macrophages, AGEs increase the expression of RAGE and pro-inflammatory cytokines (TNF-α, IL-6, IL-1β and MCP-1), induce macrophage infiltration and adhesion when co-cultivated with ECs. Interestingly, these effects are prevented by treating cells with dimethylglyoxal apigenin (DMA), a product derived from apigenin (a dietary flavonoid in celery) and MGO reaction, suggesting its potential role as therapeutic strategy to ameliorate chronic inflammatory disorders [71]. Others have previously indicated pterostilbene, a natural stilbene found in blueberries, to have an inhibitory effect on AGEs-induced oxidative stress and inflammation in macrophages, via the reduction of RAGE/MAPK/NF-ĸB pathway [72]. Moreover, retinoic acid is able to prevent the inflammatory-associated cardiac fibrosis induced by MGO administration in rats [73]. Similar results were obtained in mouse models where the overexpression of Glo1 reduces MGO-induced inflammation and prevents ventricular dysfunction [74].

### 3.5. Genomic Instability

Genomic damage is one of the hallmarks of aging cells. Throughout life, DNA integrity and stability is affected by exogenous (physical, chemical and biological agents) and endogenous (DNA replication errors, ROS and hydrolytic reactions) factors [44]. MGO directly interacts with DNA mainly reacting with the nucleotide deoxyguanosine (dG) to form the so called nucleotide AGEs, as demonstrated in human lens epithelial cells [75] and human mononuclear monocytes [16]. The major nucleotide AGE formed by MGO is the imidazopurinone derivate MGdG. Nucleotide AGEs formation increases DNA single-strand breaks and frameshift mutations [9,76]. MGO can also indirectly damage DNA by increasing ROSs, as a result of protein glycation. ROSs, in turn, interact with DNA to form 8-oxo-7,8-dihydro-2′-deoxyguanosine (8-OxodG). Both MGdG and 8-OxodG are linked to mutagenesis and cytotoxicity in aging, type 2 diabetes (T2D), renal failure and other disorders characterized by high levels of dicarbonyl metabolities [9,77]. DNA oxidative and dicarbonyl-derived nucleoside adducts are increased in plasma and urine of T2D patients and further higher in T2D patients with diabetic nephropathy (DN) [78].

In the last few years, extensive researches have been focused on the identification of strategies aimed to prevent MGO-induced DNA damage. Ferulic acid, a cinnamic acid derivative, and its isomer isoferulic acid, are able to prevent MGO-induced DNA damage through their free radical scavenging activity [79,80]. Similar protective effects are induced by cyanidin, a natural anthocyanin abundant in fruits and vegetables, that following the reduction of superoxide anion and hydroxyl radical generation is able to prevent oxidative DNA damage [81]. In diabetic rat kidneys, treatment with trans-RES (tRES) resulted in the inhibition of 8-OxodG expression in different renal components, thus ameliorating both mitochondrial and genomic DNA damage induced by AGEs accumulation and slowing down the progression of renal disease [82].

## 4. Dicarbonyl Stress in Aging-Related Diseases

### 4.1. Metabolic Disease

Metabolic disorders, including T2D and obesity, are closely related to the aging process. The global prevalence of these diseases is rapidly increasing worldwide as result of population aging, urbanization and associated lifestyle changes. An estimated 425 million people have diabetes mellitus (i.e., 1 in 11 adults), 90% of whom have T2D [83]. If present trends continue, it has been estimated that global obesity prevalence will reach 18% in men and surpass 21% in women, by 2025 [84]. Obesity itself represents a critical risk factor for the development of T2D [85]. Indeed, an increase in body fat, particularly visceral adiposity that often accompanies aging, may contribute to the development of insulin resistance, which is one of the most important risk factors for metabolic diseases in the elderly.

**Figure 2:**
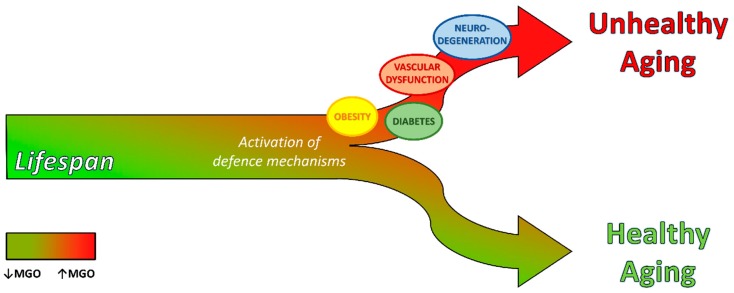
Dicarbonyl stress as determinant of aging quality. MGO accumulation during aging (red color in the arrow) induces tissue damage if not efficiently counteracted by defense mechanisms (e.g. glyoxalase system), leading to metabolic disease, vascular dysfunction, neuronal damage and, thus, unhealthy aging. If a good balance between MGO formation and defense mechanism activity is maintained, this guarantees healthy aging.

Common complications in these dis-metabolic conditions include: dyslipidemia, non-alcoholic fatty liver disease (NAFLD) and vascular dysfunction, including hypertension, coronary and peripheral artery disease, stroke and microvascular complications. Metabolic disease drastically increases the mortality risk of CVD. Therefore, there is an urgent need for enhancing our understanding of the risk factors for obesity, insulin-resistance and NAFLD to design optimal intervention to decrease incidence and health impact (Figure 2).

#### 4.1.1. Type 2 Diabetes (T2D)

Dicarbonyl stress is typical of the diabetic state and has been identified as a major contributing factor to the progression of diabetic complications [86]. The first clinical investigation on dicarbonyl stress in diabetes was a study of McLellan et al. [87], where whole-blood MGO concentrations under casual conditions were measured by high-performance liquid chromatography–mass spectrometry (HPLC/MS) and were reported to be from 3 to 4 fold higher in T2D compared to healthy patients. A similar increase was confirmed in a later study in fasting serum samples from healthy and T2D patients, by the gas chromatography–mass spectrometry (GC/MS) methodology [88]. Although LC/MS is considered the current golden standard method for MGO detection, an ELISA-based assay has just been tested and validated for quantifying MGO levels in both plasma and cell culture [89], offering a new operationally simple screening tool. A more recent investigation performed in “newly diagnosed” T2D patients demonstrated a significant 1.62 fold increase of MGO plasma levels already at early stage of diabetes in the absence of diabetic complications [90].

Dicarbonyl stress in skeletal muscle may play a causative role in the development of insulin resistance and the onset of T2D [91]. Skeletal muscle is one of the most metabolically important tissue, being responsible for more than 80% of whole body insulin-stimulated glucose disposal [92]. Because of the high glycolytic flux, skeletal muscle may be particularly susceptible to dicarbonyl accumulation when Glo1 is reduced. Glo1 expression has been recently found to be markedly reduced, together with the reduction of Nrf2 and the increase of its negative regulator Keap1, in the skeletal muscle of T2D compared to healthy control subjects. Glo1 positively correlates with glucose disposal and negatively with carbonyl stress and HOMA-IR (*HOmeostatic Model Assessment for Insulin Resistance*) [93]. Although a compensatory increase of Glo1 is able to contain a transient increase of MGO flux in metabolically healthy muscle cells, the reduced efficiency of Glo1 in metabolically compromised muscle leads to intracellular MGO accumulation. This activates molecular pathways contributing to insulin-resistance, including mitochondria damage and increased ROS production [94,95], structural changes of skeletal muscle proteins [96] and inflammation mediated through RAGE activation [97,98].

In vitro experiments performed in L6 myotubes exposed to MGO showed the inhibition of insulin-stimulated glucose uptake, likely due to the direct binding of MGO to insulin receptor substrate 1 (IRS-1), which hampers the downstream activation of insulin signaling [99]. Interestingly, exogenous MGO and Glo1 knock-down in L6 impairs glucose transporter type 4 (GLUT4) trafficking, with decreased internalization of the transporter, resulting in increased basal glucose uptake, in the absence of insulin stimulation [100]. Our studies also demonstrated the detrimental effect of AGEs on muscle insulin sensitivity both in L6 myotubes and *in vivo* in mice, through a different mechanism involving protein kinase C (PKC) activation [101,102]. *In vivo* chronic MGO administration in rodents induces metabolic changes characteristic of T2D, namely impaired glucose tolerance with reduced insulin secretion, insulin resistance and dyslipidemia [103,104]. A prolonged MGO treatment, until 12 weeks, in Balb/C mice also showed the upregulation of pro-inflammatory markers (TNF-α and IL-1β) in the liver [105].

Moreover, Francisco et al. have recently demonstrated a long-term effect of an early exposure to MGO. The maternal exposure to MGO (60 mg/kg/day by gavage) during the lactation period is able to negatively affect the rat offspring leading to T2D later in life, possibly by changes in breast milk composition. In detail, male offspring at 90 days of life show glucose intolerance, failure in β-cell function, increased body weight and dyslipidemia [106].

In vitro studies have also been performed to investigate the molecular effect of MGO on β-cell function. Initial studies on insulin-secreting cells demonstrated that at high levels (≥1 mM) MGO has a cytotoxic action [107]. We demonstrated that non-cytotoxic MGO concentrations impair both insulin action and secretion by inhibiting insulin-induced activation of insulin receptor substrate 1 (IRS1)/ phosphatidylinositol 3 kinase (PI3K)/protein kinase B (PKB), with the concomitant formation of AGE adducts on IRS, and blocking glucose-induced insulin secretion [108]. Another mechanism by which MGO can cause β-cell damage and reduce insulin-secretion, was described by Bo et al. [109]. They showed that MGO induces the activation of uncoupling protein 2 (UCP-2) in mouse insulinoma (MIN-6) cells, resulting in reduced mitochondrial membrane potential and ATP production, which can suppress glucose-stimulated insulin secretion. However, an acute exposure to MGO induces a dual effect on rat pancreatic islet function, which is an increase of insulin secretion in basal glucose concentration, but exerts the opposite effect on hyperglycemia [110]. Accordingly, in an acute stimulation MGO can act as an agonist of transient receptor potential ankyrin 1 (TRPA1) channel in β-cells, favoring Ca^2+^ influx and insulin release [111]. Therefore, MGO may have different effects in the acute and chronic exposure of β-cells, likely inducing a transient increase in insulin secretion, but compromising long-term β-cell function due to the inhibition of insulin signaling and synthesis mechanisms [112].

Besides impairing insulin secretion and sensitivity, MGO is also able to induce structural and functional changes to the insulin molecule, by attaching to arginine residue in its beta-chain. This insulin adduct impairs insulin-mediated glucose uptake in adipocytes and skeletal muscle cells, autocrine control of insulin secretion in pancreatic β-cells, and decreases insulin clearance through liver cells [113].

#### 4.1.2. Obesity

The role of dicarbonyl stress in obesity was initially suggested by a genetic linkage analysis in humans where Glo1 was liked to anthropometric measurements of obesity, and later by a meta-analysis of mice strains linking Glo1 to body weight [114,115]. A recent clinical study in obese subjects has shown an increase of 35% of plasma MGO in obese compared to overweight non-obese subjects [116]. The parallel 2-fold increase of plasma D-lactate indicates that the flux of MGO formation is increased in obesity. Obese MGO plasma levels are intermediate between those found in lean healthy controls and diabetic subjects. This has been defined a state of “moderate” dicarbonyl stress which may be, however, functionally important.

A major source of dicarbonyl stress in obesity is glyceroneogenesis. Indeed, increased fatty acid esterification occurs for triglyceride deposition during adipocyte expansion in obesity. Pyruvate represents the carbon source in glyceroneogenesis. By contrast with glucose, pyruvate has unrestricted entry and metabolism in cells susceptible to insulin-resistance, including adipocytes and hepatocytes. Thus, it represents an alternative constant source of triosephosphates [117].

During adipose tissue expansion, the interstitial oxygen tension decreases. As a result, hypoxia inducible factor 1 alpha (HIF-1α) increases in expanding adipocytes to drive adipose tissue vascularization. However, vascularization is impaired by MGO through the direct modification of the transcriptional machinery responsible for the altered vascular endothelial growth factor (VEGF)/angiopoietin-2 (Ang-2) ratio [118,119]. Angiogenesis impairment in hypoxic adipose tissue hampers an adequate tissue perfusion, thus fostering tissue dysfunction [120]. Persisting hypoxia activates stress-response pathways leading to inflammation [121]. It has been suggested that HIF-1α and NF-ĸB cooperate in the regulation of gene transcription triggering inflammatory responses during hypoxia [118]. Both HIF-1α and NF-ĸB are known to downregulate Glo1 expression. Therefore, together with the increased MGO formation from glyceroneogenesis, Glo1 downregulation by hypoxia and inflammation provide the conditions for dicarbonyl stress in obesity [116].

A cross-sectional study of obese subjects have shown a direct correlation of serum AGEs (sAGEs) with markers of inflammation and insulin resistance, and an inverse correlation between sAGEs with innate defenses (e.g. Glo1). sAGE levels in healthy obese subjects who did not have metabolic syndrome were lower than in obese subjects who met more than one criteria for metabolic syndrome (central obesity, hypertension, dyslipidemia or hyperglycemia), representing a link between healthy and unhealthy obesity [122]. Consistently, obese subjects with T2D show increased postprandial dicarbonyl stress, which is reduced by energy restriction and gastric banding, highlighting the potential for dicarbonyl reduction to prevent or delay the development of complication in unhealthy obesity [123]. Furthermore, a highly promising therapeutic intervention study has recently shown how the increased expression and activity of Glo1, induced by a tRES and hesperetin (HESP) formulation, is able to improve metabolic and vascular health in both overweight and obese subjects [124].

The molecular mechanisms of adipose tissue dysfunction under dicarbonyl stress have been explored in experimental models. MGO administration (14 weeks, 50–75 mg/kg/day) to rats leads to decreased irrigation of adipose tissue, with increased accumulation of hypoxia probe, and macrophage infiltration in glycated and fibrotic regions of adipose tissue. Similar structural alterations are also observed in the adipose tissue of aged rats [125]. Adipose tissue hypoxia was confirmed in rats under high fat diet (HFD) supplemented with MGO, but not under HFD alone. Hypoxia was associated to impaired adipose tissue blood flow, hampering its expandability during HFD and leading to insulin resistance [126]. In detail, adipose tissue from MGO-treated rats shows an imbalance of VEGF/Ang-2 ratio in parallel to increased levels of CD31, suggesting a compensatory EC proliferation and formation of aberrant capillaries. Furthermore, MGO supplementation to HFD caused systemic dysmetabolism, as indicated by higher free fatty acid (FFA) levels, insulinemia, glucose intolerance and insulin resistance, demonstrated by reduced insulin receptor, Akt activation and GLUT4 levels in skeletal muscle [126]. MGO accumulation was previously reported to impair insulin-stimulated glucose uptake, plasma membrane GLUT4 expression, and PI3K activity in both adipose tissue of rats and 3T3-L1 adipocytes [103,127].

Through this sequence of events, MGO-induced glycation may lead to the onset of unhealthy obesity and T2D progression.

### 4.2. Vascular Dysfunction

CVD and, more generally, vascular dysfunction, are prevalent conditions in the elderly, often associated with metabolic alterations. A new concept of early vascular aging (EVA) has developed in the last decade, and encompasses early vascular changes at different layers of the vessel wall, representing the origin of several tissue dysfunction [128]. Aging and other factors, including diabetes and obesity, can cause disease of the heart (CVD), brain (cognitive disease) and peripheral tissues (impaired angiogenesis and tissue perfusion, insulin resistance, microvascular complications) through vascular dysfunction (Figure 2). The latter includes dysfunction of large arteries, microcirculation and endothelium [129].

Experimental data have shown that dicarbonyl stress contributes to the impairment of both micro- and macro-vascular dysfunction, also independently of hyperglycemia [12].

#### 4.2.1. Microvascular Dysfunction

Angiogenesis is a multistep process crucial for reperfusion and wound healing in damaged tissues. Accumulating evidence indicates that physiological angiogenesis is compromised by MGO. Although high MGO levels lead to the formation of aberrant capillaries in zebrafish by upregulation of vascular endothelial growth factor receptor 2 (VEGFR2) [130], ECs exposed to MGO show impaired viability, migration [131] and tube formation through RAGE-mediated and autophagy induced VEGFR2 degradation [132]. As mentioned above, MGO interference with gene transcription induces an imbalance of VEGF/Ang-2 ratio, thus compromising an adequate tissue perfusion [120], also leading to EC apoptosis and increased vessel permeability in the retina [119]. The suggestion deriving from the existent studies is that the dysregulation of VEGF action by dicarbonyl stress sustains aberrant angiogenesis. In line with this, the overexpression of Glo1 inhibits AGEs formation in ECs [133], favors both angiogenesis *in vitro* [134] and *in vivo* facilitating wound healing [135] and muscle reperfusion after ischemic insults in diabetic rats [136]. Our group has recently identified the antiangiogenic factor Homeobox A5 (HoxA5) as a new player in MGO-induced angiogenesis impairment of ECs knock-down for Glo1. HoxA5 overexpression is regulated by NF-ĸB-p65, which is activated by MGO accumulation [137].

MGO dependent activation of NF-ĸB, followed by apoptosis, was also found in primary pericyte cultures and the lens of diabetic rats [138,139]. Moreover, MGO suppresses the proliferation and induces cell death through mitochondrial dysfunction in retinal pigment epithelial cells [52,140]. Accordingly, together with the alteration of cell adhesion properties [141], MGO contributes to the loss of retinal vessel integrity. *In vivo* exogenous administration of MGO recapitulates diabetic retinopathy-like changes, including pericytes loss, formation of acellular capillaries and early neuronal dysfunction [142]. Furthermore, gain and loss of function studies with Glo1 reveal its modifier role in the development of retinal damage, also in the absence of chronic hyperglycemia [143,144]. Importantly, the occurrence of diabetic retinopathy has been correlated with serum levels of MG-H1 in diabetic subjects [145].

Dicarbonyl stress and increased formation of MG-H1 represent key contributory factors of chronic kidney disease (CKD), likely as result of Glo1 downregulation in non-diabetic subjects [146]. This has also been experimentally proven by the development of DN-like changes in Glo1 knock-down non-diabetic mice [147]. Moreover, MGO accumulation in Wistar rats impairs several renal disease markers progressively observed in diabetic Goto–Kakizaki rats [148]. Glo1 downregulation also represents a common feature of experimental DN in response to HIF-1α and inflammatory pathways interfering with Nrf2 activity. Conversely, Glo1 overexpression is able to prevent age-related decline in renal function and DN [149,150]. In humans, a correlation between MGO-derived AGEs and early disease progression was observed in diabetic patients [151], recently confirmed by higher levels of GO and MGO found in T2D patients with DN compared to those without DN [152]. Furthermore, an association study between renoprotective factors and CKD progression has revealed a positive correlation of the dicarbonyl and L-xylulose reductase (DCXR) expression with dicarbonyl stress-detoxifying enzymes [153].

#### 4.2.2. Macrovascular Dysfunction

Besides microvascular complications, increased levels of MGO have also been associated with the development of hypertension and atherosclerotic processes. A recent integrative genomic study revealed Glo1 deficiency as a driver of CVD [154].

Impaired endothelium-dependent vasorelaxation represents a common feature of endothelial dysfunction and is an important cause of hypertension, particularly in elderly people [155,156]. Among the bioactive molecules generated by the endothelium, nitric oxide (NO) plays a pivotal role in vascular homeostasis. We and others have demonstrated an impairment of endothelium-dependent NO release and vasorelaxation by MGO.

MGO accumulation prevents the insulin-dependent activation of the IRS1/Akt/endothelial nitric oxide synthase (eNOS) pathway, thereby blunting the NO production in response to insulin both in aortic tissue *in vivo* and in ECs *in vitro*, while extracellular signal-regulated kinase (ERK1/2) activation and endothelin-1 (ET-1) release is increased by MGO [103]. We further identified the down-regulation of miR-190a and miR-214 as an epigenetic mechanism induced by MGO, contributing to endothelial insulin-resistance through the regulation of the kinase kirsten rat sarcoma viral oncogene homolog (KRAS) [157] and the Akt phosphatase PH Domain And Leucine Rich Repeat Protein Phosphatase 2 (PHLPP2) [158], respectively.

Ex vivo exposure to MGO reduces endothelial nitric oxide synthase (eNOS) activity, NO production and NO-dependent vasorelaxation of isolated arteries [159,160]. This effect is prevented by MGO scavengers aminoguanidine (AG) and N-acetyl-cisteine (NAC) [161], and by Glo1 over-expression [162]. Chronic MGO administration induces endothelial dysfunction in Wistar rats and aggravates the endothelial dysfunction developed by diabetic Goto–Kakizaki rats, through increasing oxidative stress, AGE formation, RAGE expression and inflammation [163]. Furthermore, the RAGE-mediated activation of NF-ĸB increases the renin-angiotensin levels and blood pressure in rats treated with MGO [164]. At vascular smooth muscle cell level, MGO modifies ion channel structure, which results in acute changes in vessel tone but long-term inhibition that hinders vessel relaxation [165,166].

A vascular glycation effect on the endothelial function in aging was investigated by Jo-Watanabe et al. in Glo1 transgenic rats, showing that Glo1 prevents the age-related impairment of endothelium-dependent vasorelaxation due to NOS inactivation and decreased NO production [167].

MGO has also been involved in the development of atherosclerotic plaque. Tikellis et al. demonstrated that increasing plasma MGO to levels observed in diabetic mice is sufficient to increase vascular adhesion and atheroneogenesis in normoglycemic apoE^-/-^ mice to a similar extent as that observed in diabetic mice [168]. This MGO-mediated effect has been attributed, at least partly, to RAGE activation. The latter promotes atherosclerosis by triggering intracellular pathways that lead to the expression of cytokines, cellular adhesion molecules, ROS and the vascular matrix metalloproteinases [169,170]. Conversely, RAGE deletion attenuates atherosclerosis and vascular inflammation associated with diabetes, induced in apoE^-/-^ mice [167]. MGO administration to rats induces dyslipidemia [171]. It modifies low-density lipoproteins (LDL) favoring their atherogenicity, by increasing molecular density and binding to proteoglycans in the arterial wall [172]. Levels of MGO-glycated apolipoprotein B100 of LDL are increased up to 5-fold in T2D patients [173]. Furthermore, MGO modification of high-density lipoproteins (HDL) restructures the HDL particles, increasing density, decreasing stability and their plasma half life [174].

In humans, high levels of MG-H1 residues were found in carotid atherosclerotic plaques associated with a rupture-prone phenotype [175], and have been proposed as a marker of early atherosclerotic stages in childhood diabetes [176]. MGO-derived AGEs were also correlated with increased aortic stiffness, a hallmark of aging and atherosclerosis, in an adult population [177]. More recently, higher plasma MGO levels have been associated with incident CVD in type 1 diabetes (T1D) and with cardiovascular mortality in T2D [178,179], while Glo1 activity is reduced in the atherosclerotic lesion of non-diabetic patients with increased Glycated hemoglobin (HbA1c) [180]. The clinical study by Xue et al. [124] in healthy overweight and obese subjects has demonstrated that the increased activity of Glo1, induced by the tRES-HESP formulation, is able to improve arterial dilation and decrease vascular inflammation.

These studies suggest that dicarbonyl stress represents a relevant contributing factor for vascular aging and CVD through the alteration of vascular homeostasis and dyslipidemia.

#### 4.2.3. Acute Disease

Beside the described implications of dicarbonyl stress on the progression of chronic vascular complications, some studies have revealed an association between MGO and the aftermath of acute injuries. MGO and carboxymethyl-lysine (CML) were found to be increased in the serum of patients after an acute trauma, and persistently elevated levels (over 2 weeks from the trauma) were associated with a greater severity of injury [181]. Investigations in an Indian population showed a significant increase of MGO in acute renal failure induced by snake venom [182], also highlighting MGO as an independent predictor of poor prognosis in snakebite-induced acute kidney injury [183]. Twenty-four hours after transient renal ischemia, increased MGO adducts were found in rats and Glo1 overexpression decreased the damage after ischemia [184]. Furthermore, the disturbance of energetic balance with increased MGO formation also follows severe acute neuronal lesions and both Glo1 expression and cellular localization change following brain ischemia [185,186].

These studies reveal that pathological mechanisms described for chronic complications during aging, i.e., hypoxia, oxidative stress and increased inflammation, also appears to play a role in trauma-related organ failure characterized by increased dicarbonyl stress [12]. Although similar mechanisms are reported to be activated in acute injury and chronic progression of tissue damage, present clinical studies are still not exhaustive enough to state whether dicarbonyl stress associated with acute injury is crucial in accelerating aging. This is partly due to the short duration of the follow-up. Conversely, it is clear that the aging-dependent impairment of defense mechanisms lowers the host response to acute hypoxic-ischemic injury.

### 4.3. Neurodegeneration

Increasing age is undoubtedly associated with cognitive impairment and chronic neurodegenerative diseases, such as Parkinson’s (PD) and Alzheimer’s (AD) diseases [187]. These clinically distinct diseases share multiple mechanistic similarities. Common underlying mechanisms responsible for PD- and AD-associated neurodegeneration are abnormalities in protein folding and aggregation [188], progressive loss of neuronal cells [189], increased oxidative stress and inflammation [190]. In particular, extracellular amyloid β-peptide (Aβ) deposits (amyloid plaques) and intracellular tau protein precipitates (neurofibrillary tangles) are pathological hallmarks of AD and Lewy bodies, cytoplasmic inclusions mainly composed of α-synuclein (α-syn), characterize PD. 

Reactive dicarbonyls are unquestionably relevant players in PD and AD pathogenesis, due to their ability to directly damage neurons, forming crosslinks and AGE adducts, increasing ROS production and inducing apoptosis [191]. In frontal cerebral cortex of human brains with AD, carboxyetil-lysine (CEL) and CML levels are elevated [192]. N-epsilon(carboxymethyl)lysine and 3-deoxyglucosone-derived hydroimidazolone levels are elevated in cerebrospinal fluid (CSF) protein of AD patients. In CSF ultrafiltrate, the concentrations of MGO-derived hydroimidazolone and GO-derived hydroimidazolone-free adducts were augmented, as well. Histochemical analysis demonstrated that AGEs and RAGE levels were increased in the frontal cortex of PD patients compared to controls [193]. Furthermore, immunoreactivity to pentosidine and pyrraline, two specific AGEs, was seen in Lewy bodies in the substantia nigra of PD patients [194] and AGEs have been shown to be inducers of both protein crosslinking and free radical formation [195]. Interestingly, a negative correlation between a decline in cognitive function assessed by the MMSE (mini-mental state examination) and fructosyl-lysine levels was found [196].

#### 4.3.1. Glycation Targets in Parkinson’s (PD) and Alzheimer’s Disease (AD)

Due to its stability, Aβ represents an ideal substrate for non-enzymatic glycation leading to AGEs formation. In addition, MGO is associated with enhanced aggregation of Aβ in MC65 human neuroblastoma cells [197] and AGEs regulate amyloid precursor protein (APP) processing and Aβ accumulation [198]. In more detail, AGEs up-regulate APP gene and protein through a ROS mediated mechanism and enhance the production of Aβ [199], upregulating BACE (beta-site APP-cleaving enzyme-1) and PS1 (presenilin 1) expression. Interestingly, Aβ is a ligand of RAGE [200] and treatment of hippocampal neurons with Aβ-AGE increases RAGE expression. Moreover, glycation confers an altered secondary structure to Aβ enhancing its affinity for RAGE and the downstream activation of glycogen synthase kinase 3 (GSK-3), one of the principal mediators involved in AD pathogenesis [201]. It has been shown that Aβ-AGE is more effective than Aβ in decreasing cell viability, inducing apoptosis and tau hyperphosphorylation and reducing synaptic proteins [201].

As observed in AD for Aβ, glycation exacerbates α-syn toxicity and aggregation in human cell lines and in differentiated patient-derived iPSCs (induced pluripotent stem cells). It is worth noting that in iPSCs the cytotoxic effects of MGO are abolished when α-syn is knocked down, confirming that MGO cytotoxicity depends on α-syn levels [202]. In α-syn transgenic *Drosophila*, glycation worsens the motor performance and decreases longevity. Similarly, in α-syn expressing mice, MGO injection in the substantia nigra causes a significant loss of neuronal cells, including TH-positive neurons. Moreover, in brain tissue from wild-type or from human α-syn BAC transgenic mice, the glycated α-syn amount increases with age. Importantly, glycation affects α-syn physiology at different levels. Indeed, it promotes both α-syn oligomerization and formation of α-syn inclusion in cell models of PD. In particular, glycation modifies the N-terminal structure of α-syn, impairing its ability to bind to lipid membranes and reduces its clearance altering the ubiquitin proteasome system and the autophagy lysosome pathway [202]. Beside α-syn, another important glycation target related to PD pathogenesis is dopamine itself. It has been shown that MGO can react with dopamine, generating a potentially toxic product (1-acetyl-6, 7-dihydroxy-1, 2, 3, 4-tetrahydro-isoquinoline, ADTIQ) whose structure strongly resembles that of 1-Methyl-4-phenyl-1, 2, 3, 6-tetrahydropyridine (MPTP), commonly used to induce experimental PD. Interestingly, ADTIQ has been found to accumulate in PD brains [203].

#### 4.3.2. Dicarbonyl Stress and Cognitive Decline

Dicarbonyl stress in older people also actively participates in cognitive decline, which characterizes both PD and AD, leading to a further worsening of life quality. The relationship between circulating levels of MGO derivatives and cognitive decline over time has been investigated in 267 very elderly non-demented subjects. Increased rates of cognitive decline have been found to be associated with higher MGO serum levels [204]. Similar results were obtained by another study, performed in 378 participants aged between 60 and 85 years, where a higher MGO serum amount was associated with poorer memory and executive function and with lower grey matter volume [205]. Interestingly, an association between declining cognitive speed and urinary AGE pentosidine levels in old subjects was observed too [206]. Indeed, old adults with high urine pentosidine feature worse baseline DSST (digit symbol substitution test) score but similar 3MS (Modified Mini-Mental State Examination) score. Furthermore, CML staining in cortical neurons and cerebral vessels is correlated to the severity of cognitive impairment in individuals with cerebrovascular disease, confirming that AGEs play a role in cognitive dysfunction in cerebrovascular disease [207]. The relevance of MGO and AGEs in cognitive function has emerged also from studies performed in animal models, which allowed to clarify the molecular mechanisms underlying their deleterious action. Indeed, inhibition of AGEs can revert the cognitive deficit in AD-like mice models [201]. Chen et al. showed that Aβ-AGE, stereotactically injected into lateral ventricle of Sprague–Dawley rats, worsen Aβ-induced cognitive impairment, characterized by higher speed of deterioration of long-term potentiation (LTP), decreased dendritic spines density and increased down-regulation of synaptic proteins. These phenomena are accompanied by RAGE overexpression and by the consequent activation of RAGE downstream molecular effectors (GSK3, NF-κB, p38). Cognitive decline amplified by Aβ-AGE was reverted by simultaneous application of the RAGE antibody or GSK3 inhibitor lithium chloride (LiCl) [208].

#### 4.3.3. Methylglyoxal (MGO) Detoxification and Neurodegeneration

Insufficient detoxification of MGO by the glyoxalase system significantly contributes to dicarbonyl accumulation observed in neurodegeneration. Indeed, it has been shown that alterations of the glyoxalase system directly impact on AD severity [209] and Glo1 expression levels are increased in a compensatory manner in an early stage but decreased in a late stage of AD [1]. Similarly, PD is characterized by a marked decrease of cellular amount of reduced GSH which leads to a lower glyoxalase system activity [190]. The gene DJ-1/ Parkinson disease protein 7 (PARK7), involved in the onset of Parkinson’s disease, belongs to a novel glyoxalase family and influences mitochondrial activity and oxidative stress [210]. Interestingly, glycated α-syn is a direct substrate for DJ-1 deglycase activity and DJ-1 overexpression reduces MGO-induced aggregation of α-syn in Neuro-2A cells [211]. Importantly, Glo1 inhibition reduces neuronal survival and induces accumulation of AGEs, while Glo1 overexpression reduces ROS amount [212].

Thus, the glyoxalase pathway is a crucial antioxidant defense mechanism against aging-associated neurodegeneration [213] and increasing glyoxalase pathway efficiency represents a promising strategy to reduce the severity of both aging and neurodegenerative disease (Figure 2). Recently, flavonoids, secondary plant metabolites commonly present in fruits and vegetables [214], have been found to own antioxidant properties, due to the ability to scavenge ROS directly and to improve glyoxalase pathway activity. In particular, flavonoids enhance the expression of γ-glutamyl-cysteine synthetase (GCS) subunits mRNA [215], leading to an increase of intracellular GSH concentration and to a reduction of MGO levels [216]. In addition, treatment with flavonoids augments Glo1 and Glo2 expression levels in MGO treated cerebellar neurons, attenuating MGO induced neurotoxicity [216]. These studies confirm that glyoxalase pathway is a promising drug target for treatment of neurodegenerative diseases and pave the way for innovative studies focused on design, synthesis and testing of more efficient flavonoids.

## 5. Concluding Remarks

Dicarbonyl stress is associated with metabolic and age-related diseases. Not only do dysmetabolic conditions and aging favor the formation and accumulation of dicarbonyls, but increasing reports sustain a causal role of dicarbonyl stress itself in cellular aging and tissue dysfunction. Indeed, when cellular defenses fail to counteract dicarbonyls increase, a plethora of pathological molecular pathways get started, leading to metabolic complication, vascular dysfunction, aging and neurodegeneration. These conditions compromise healthy aging and represent a serious burden on public healthcare expenditures.

Extensive longitudinal studies are needed to prove whether elevated dicarbonyl levels may be predictive of unsuccessful aging. This can be achieved by the optimization of operationally simple screening tools and the setup of population-based studies. Although the activation of several mechanisms contribute to the onset of aging-related disease, proving that dicarbonyl stress is a common trigger of these pathological pathways will sustain the potential for dicarbonyls-targeted pharmacological strategies to overcome the progression of aging-related diseases. Therefore, further efforts are needed to optimize clinical intervention to alleviate dicarbonyl stress and guarantee healthy aging.
